# Understanding the effects of socioeconomic status along the breast cancer continuum in Australian women: a systematic review of evidence

**DOI:** 10.1186/s12939-017-0676-x

**Published:** 2017-10-16

**Authors:** Greg Lyle, Gilly A. Hendrie, Delia Hendrie

**Affiliations:** 1 Centre for Population Health Research, Curtin University, Perth, Australia; 2 CSIRO Health and Biosecurity, Adelaide, Australia; 3 School of Public Health, Curtin University, Perth, Australia

**Keywords:** Breast cancer continuum, Socioeconomic status, Feedback diagram, Australia

## Abstract

**Background:**

Globally, the provision of equitable outcomes for women with breast cancer is a priority for governments. However, there is growing evidence that a socioeconomic status (SES) gradient exists in outcomes across the breast cancer continuum – namely incidence, diagnosis, treatment, survival and mortality. This systematic review describes this evidence and, because of the importance of place in defining SES, findings are limited to the Australian experience.

**Methods:**

An on-line search of PubMed and the Web of Science identified 44 studies published since 1995 which examined the influence of SES along the continuum. The critique of studies included the study design, the types and scales of SES variable measured, and the results in terms of direction and significance of the relationships found. To aid in the interpretation of results, the findings were discussed in the context of a systems dynamic feedback diagram.

**Results:**

We found 67 findings which reported 107 relationships between SES within outcomes along the continuum. Results suggest no differences in the participation in screening by SES. Higher incidence was reported in women with higher SES whereas a negative association was reported between SES and diagnosis. Associations with treatment choice were specific to the treatment choice undertaken. Some evidence was found towards greater survival for women with higher SES, however, the evidence for a SES relationship with mortality was less conclusive.

**Conclusions:**

In a universal health system such as that in Australia, evidence of an SES gradient exists, however, the strength and direction of this relationship varies along the continuum. This is a complex relationship and the heterogeneity in study design, the SES indicator selected and its representative scale further complicates our understanding of its influence. More complex multilevel studies are needed to better understand these relationships, the interactions between predictors and to reduce biases introduced by methodological issues.

## Introduction

Addressing the delivery of equitable health services for the identification and treatment of cancer is a priority for high income nations. In Australia, like many other industrialised countries, breast cancer is the most common cancer affecting women [1]. A considerable amount of resources are directed to address issues relating to breast cancer along a continuum; i.e. the prevention, incidence/risk, detection/diagnosis, treatment, survivorship, survival and mortality [2]. However, there is growing evidence of socioeconomic status (SES) gradients along the stages of the breast cancer continuum [3–7]. The personal impacts such as reduced quality of life and premature deaths; societal impacts such as lost productivity; direct and indirect health care costs of these disparities are substantial and need to be addressed.

Past reviews [5, 8] have highlighted methodological issues which need to be considered to interpret the literature. The definition and measurement of SES is variable between studies, however, SES is usually measured through the socioeconomic triad, a mixture of single or composite indicators of employment, income and education. These indicators have been found to have varying associations depending on the type of cancer investigated [9]. The scale at which these SES indicators are collected has been found to influence these relationships. SES data collected through surveys from individuals represent what are seen as compositional factors which either promote or impede an individual’s health [10, 11]. For example, individuals reporting higher levels of education [12] and income [13] have been found to have higher incidence of breast cancer but a lower risk of mortality. Where individual level data is not available, area level SES measures collected from national surveys or statistics have been used to represent two different perspectives. One is to mimic, at an aggregated level, the compositional individual level SES factors. Several studies have found mixed findings as to how well these area based statistics reflect individual circumstances [14–17]. The second has been to embody the contextual factors in which the individual resides [11], providing additional information on living circumstances not captured by individual level variables [14]. These represent differences in the physical environment, service provision, as well as political and cultural characteristics which, independent of personal circumstances, can influence health behaviour and health status [2, 10, 18]. Recent reviews have shown modest positive associations between the SES of women’s residential area and breast cancer incidence and screening but not mortality [4, 19]. However, methodological differences relating to the scale of analysis used and the type of SES indicator used have led to mixed findings. For example, SES derived in smaller geographic units compared to larger units has produced stronger relationships in breast cancer survival [20] and incidence [21]. While the choice of indicator used and how it was defined (single versus a composite indicator) have also impacted the direction and magnitude of associations with survival [22]. Where individual and area level SES data are available, several studies have undertaken investigations into each characteristics’ effects, reporting a higher incidence [23] and survival [24] from breast cancer in individuals with higher levels of education or woman living in areas of higher SES. To further evaluate the interactive effects between individual and area level characteristics, multilevel analysis has been used to disentangle the compositional and contextual level influences for specific points along the continuum. Several studies have reported a positive relationship between area level SES and incidence of breast cancer [23, 25] while others report no association [26, 27] citing individual SES as the major determinant [27]. These analyses overcome several methodological limitations present in looking at individual and area level SES associations independently, namely, individual and ecological fallacies and the Simpsons Paradox [28, 29].

Differences in the way SES is conceptualised, operationalised and analysed make comparisons between studies, and coming to a consensus, difficult. However, these must be taken into account so that the evidence base can inform future research in these areas and government policy. The aim of this study was to conduct a systematic review of literature to gather a weight of evidence to determine the strength and direction of the SES relationships across the breast cancer continuum. Given that the concept of place is fundamental to understanding these relationships, we will restrict this review to the Australian experience. This removes issues which relate to studies based in different countries where there are differences in the delivery of health services (universal versus private) and differences in the racial and ethnic structure of the population. Furthermore, by limiting these studies to one geographic area, we can investigate the effect of methodological issues on the relationships and highlight any gaps in our understanding. In our review we also restrict the definition of the breast cancer continuum to incidence, detection, diagnosis, treatment, survival and mortality. This allows us to focus on specific areas of outcomes and care. To tie the evidence base across the continuum together we discuss the findings through a simple systems dynamics feedback diagram to provide a deeper understanding of the interacting processes involved in incidence, detection, diagnosis, treatment and their effect on the outcomes of survival and mortality. Gathering evidence at each stage, determining the direction of significant trends, highlighting evidence gaps for researchers, and understanding potential feedbacks between and within the stages will help to guide delivery of care and improve equity in outcomes which remains a considerable challenge for policy makers.

## Methods

### Search strategy

A list of search terms and keywords was developed and refined to reflect a focus on the socioeconomic inequalities across the breast cancer continuum among Australian women. Search terms lists were comprehensive and inclusive, combined under the following headings: socioeconomic status, breast cancer and Australia. Search terms were combined as follows:

1. Socio-economic status: e.g. SES, socio-economic, socioeconomic, disadvantage, deprivation, income, poverty, education, and employment; AND.
2. Breast cancer; AND.
3. Australia

The search was conducted at the end of July 2016 and included PubMed and the Web of Science databases. Studies were included if they met the inclusion criteria (described below). Unpublished work or published protocols without results were not included in the search. Grey literature were not included in the review as not all are indexed in scientific databases, however the relevant government reports we could access were included in the discussion. Finally, reference lists of identified articles were searched for additional relevant studies.

### Study inclusion criteria

#### Types of participants

Australian female adults were the primary interest group for this review; therefore studies of adult (aged 18+ years) female members of the general Australian population were included.

#### Types of studies

Studies that examined the influence of SES (predictor) on breast cancer (outcome) were included. SES could be measured at the individual level (such as income or education), at an area level (such as indicators of socioeconomic status) or a combination of both. Studies of any design were included such as secondary analysis of population cancer registries, cross sectional retrospective telephone surveys and other cohort studies.

#### Types of outcomes

From the selected studies we extracted evidence of the types of findings which related to whether individual, area, individual and area or multilevel analyses were used and the significance of the relationships found (positive, negative, non-significant or non-reported) of SES gradients across the breast cancer continuum. This methodology took into account the hierarchical nature of the studies undertaken. Here, a study could potentially have multiple types of findings using a variety of individual and area level SES indicators and then report numerous significant results of varying directions. Direction of significance was determined using higher socioeconomic position/least disadvantage as the reference position. Therefore a positive relationship described when a higher socioeconomic position was associated with a higher incidence, higher likelihood of screening or treatment, or greater survival.

### Study exclusion criteria

To limit the focus to socioeconomic inequalities in the general Australian adult population, studies that examined specific subgroups of the population such as Indigenous groups, children, individual case studies, people in specific workplaces, or males were excluded. We recognise that Indigenous women are a specific subgroup of the population who are more likely to be lower SES and experience poorer health outcomes. However, given the complex interaction of personal, social, and cultural factors affecting Indigenous women, and the generalisability of findings, the studies addressing this subgroup specifically were excluded from this review.

We limited our search to incidence, detection, diagnosis, treatment, survival and mortality along the breast cancer continuum. Studies that focused on the behavioural risk factors of cancer such as diet and lifestyle were excluded, as were those focused on the psychosocial predictors, such as knowledge and attitudes, towards cancer related behaviours or treatment. Studies retrieved in the search that controlled for socioeconomic factors as a covariate within their analysis but did not include it as an independent variable were also excluded.

**Fig. 1:**
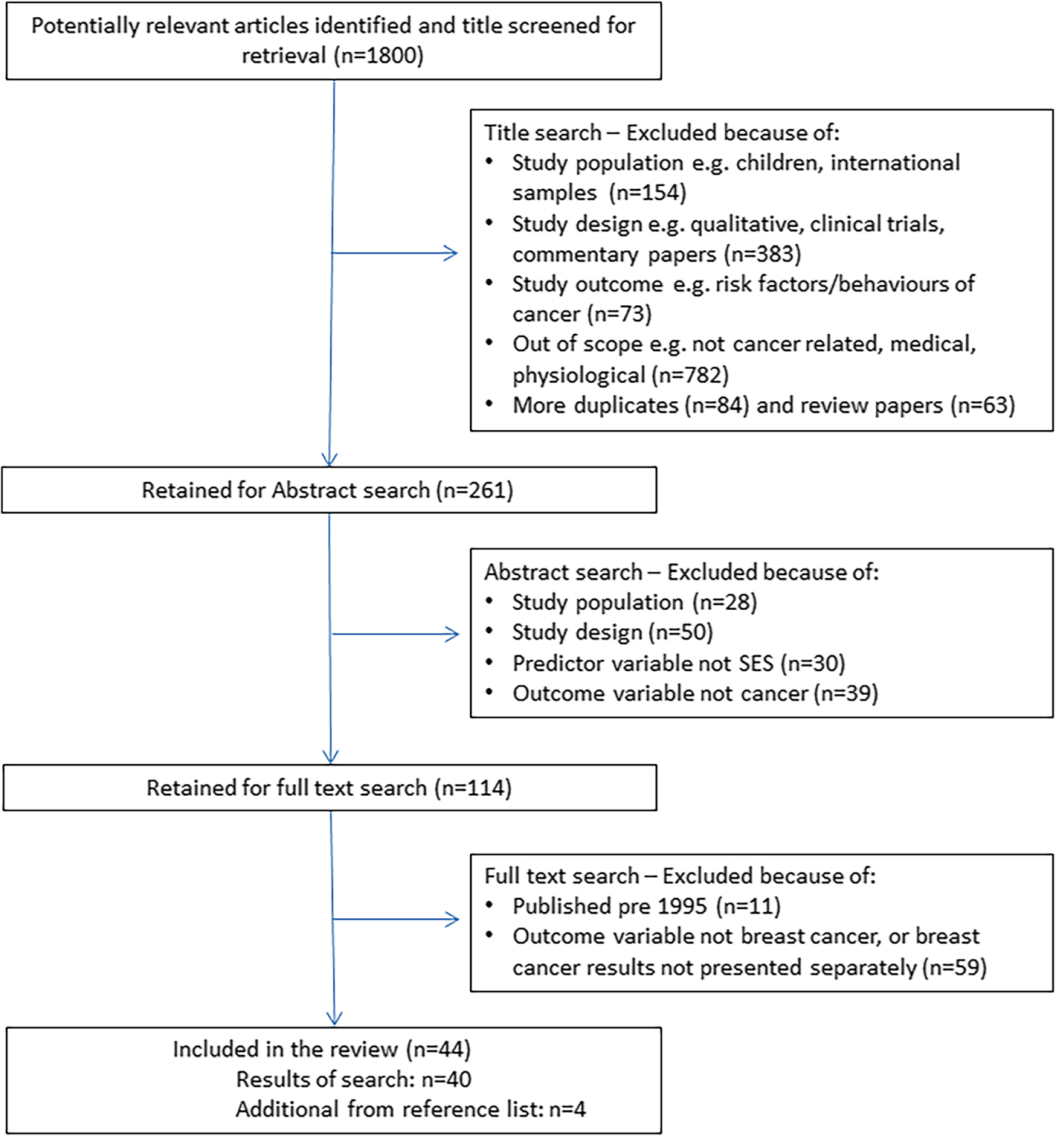
Quorum statement flow diagram: Studies examining the influence of SES across the breast cancer continuum in Australian women

Studies were limited to those published in English in the last 20 years (1995 onwards), to ensure that the findings of this review are relevant to the current healthcare context in Australia. One reviewer screened titles against inclusion criteria for eligible articles, then two reviewers screened abstracts and full text against the inclusion criteria with the final decision for inclusion made by the first author (Fig. 1). A total of 44 papers were identified through the search terms and from the reference lists of the studies collected.

### Study quality assessment

We have assessed the body of evidence in relation to a socioeconomic gradient in breast cancer across the continuum in Australian women. Some of the protocols for assessing the quality of evidence are directed more towards biomedical literature reviews (e.g. PRIMSA [30]) or clinical intervention (e.g. EPHPP [31]) which are not appropriate for this review. The National Health and Medical Research Council of Australia suggests five key components for rating the ‘body of evidence’: the evidence base, consistency of results, clinical impact, generalisability of results, and applicability of results [32]. The depth of the evidence base and consistency of its results will form the basis of the quality assessment for this review as these provide a picture of the internal validity of the study data that will support the development of policy. The other components of generalisability and application to the Australian Healthcare context are not really applicable here given this review is limited to studies of Australian women, and therefore highly generalisable and relevant to the target population. As part of this review we provide comment on the quantity of evidence for each stage across the continuum, that is the number of studies and consistency of findings, and the type of analysis undertaken in order to control for bias, for example where univariate or multivariate analysis was conducted. Relevant details were extracted from the papers as part of the data extraction process, checked by two reviewers and discrepancies resolved through discussion. Interpretation of the study quality was integrated into the description of results and agreed to by all authors. The majority (81%) of studies analysed population based datasets of state or the whole Australian population meaning that sample sizes were large and results representative of the Australian population more generally. When the evidence was from cross sectional studies, such as telephone surveys, the number of such studies was articulated within the stage of the continuum. While the quality of these studies could be deemed inferior to population studies of larger samples, a trade-off exists because these studies provide valuable information about individual level SES, which is not usually collected in larger population studies. Therefore, the study findings were not weighted, but their results discussed within the body of evidence for each stage along the continuum.

### Data extraction strategy

Using a standardised Excel spreadsheet, one reviewer extracted data from papers including details of the study population, study design, dataset analysed i.e. name of the cancer or screening registry, used, stage across the continuum, types of socioeconomic variables used at individual and area levels and the scale at which the socioeconomic variable was measured (individual, area, individual and area, multilevel), statistical analysis, results and summary of findings. A second reviewer checked all data extracted, and the few discrepancies were resolved through discussion. Given the heterogeneity in measurement of SES, cancer outcomes and statistical analysis conducted, meta-analysis was not possible. Instead the data synthesised using frequency counts and described as a narrative of findings.

## Results

### Overview

A total of 44 studies were included in this review. Around a third of the studies were conducted in cohorts from the New South Wales Cancer Registry. Seven studies were from Western Australia and an additional seven studies analysed national datasets. From these 44 studies, 67 findings were extracted describing the impact of SES across the continuum – incidence, diagnosis, treatment, survival and mortality. Twelve findings (18%) measured SES using individual level indicators thereby highlighting compositional factors only, 31 (46%) measured SES with area level indicators highlighting a mix of either compositional or contextual factors. Twenty-one findings (31%) measured SES at individual and area levels separately while three studies (4%) conducted multilevel analysis. From these types of findings we found a total of 51 individual level and 56 area based SES indicators were used to quantify 107 relationships between SES and outcomes along the continuum. To quantify individual level SES, private health insurance membership (*n* = 21, 41%) was most commonly used, followed by level of education (*n* = 12, 24%), employment status (*n* = 12, 24%) and income (*n* = 6, 12%). Across the continuum, individual level associations were dominated by non-significant results particularly for employment and education status. However, there was a weak positive association between private health insurance membership and higher income and more positive breast cancer outcomes (results not shown). For area level SES, the most commonly used indicator (*n* = 42, 76% of area level studies) was the Index of Relative Socioeconomic Disadvantage (IRSD). The index relates to the degree of area based social disadvantage based on low levels of income and education and high unemployment [33]. This composite indicator is provided nationally by the Australian Bureau of Statistics under their Socio-Economic Indexes for Areas indicator program. Income, education and employment information as separate single indicators were less frequently used. The scale of area based analysis varied from fine through to coarse levels. In terms of size and how many times used, these varied by population from lowest to highest: the Collector District (CD) (*n* = 15, 25%), postcode (*n* = 11, 20%), Local Government Area (LGA) (*n* = 11, 20%), Statistical Local Area (SLA) (*n* = 10, 18%). Eight findings (15%) were from an area which was not defined and one used a municipality boundary.

At an aggregated level, the directions of relationships between SES and outcomes in the continuum were similar but this depended on how the research question was phrased and its effect within each stage of the continuum. A more detailed investigation at each stage is provided in the next sections.

### Screening

Fifteen findings described the impact of SES on breast cancer screening (Table 1). The majority of studies were telephone interviews or surveys which were cross sectional in nature. Given these study design characteristics, individual factors of SES such as education, income, employment and private health insurance and its impact on attendance at screening were mainly investigated. From these 15 findings, 32 SES relationships were identified (Table 1), half the relationships showed non-significant relationships between SES and screening, but this was dependent on what specific type of screening participation was investigated. Therefore we have described the results separately as those that have never participated in screening, recent participation and overdue screening. Several studies reported on combinations of these situations.

**Table 1:** The types of findings and number and direction of significance of SES relationships across the breast cancer continuum

	Types of findings					Significance			
Stage	Individual	Area	Individual and area	Multilevel	Total	Positive	Negative	NS^	NR#
**SCREENING: TOTAL**	**11**	**2**	**2**		**15**	**6**	**10**	**16**	
**Screening - Never**	**2**		**1**		**3**	**1**	**3**	**4**	
Individual	2					1	2	4	
Area - Not defined			1				1		
**Screening - Recent**	**6**				**6**	**2**	**2**	**9**	
Individual	6					2	2	9	
**Screening - Overdue**	**3**	**1**	**1**		**5**	**3**	**4**	**3**	
Individual	3					3	3	2	
Postcode		1						1	
Area - Not defined			1				1		
**Screening - access to services**		**1**			**1**		**1**		
CD		1					1		
**INCIDENCE: TOTAL**		**5**			**5**	**5**	**1**		
CD		1				2			
LGA		3				2	1		
SLA		1				1			
**DIAGNOSIS: TOTAL**		**5**	**4**	**1**	**10**	**2**	**8**	**4**	
**Stage of diagnosis**		**5**	**1**	**1**	**7**		**7**	**3**	
Individual							1	1	
CD		2	1				2	2	
Postcode		1					1		
LGA		1					1		
SLA		1		1			2		
**Diagnostic procedure**			**1**		**1**	**1**		**1**	
Individual								1	
Area - Not defined			1			1			
**Diagnostic results**			**2**		**2**	**1**	**1**		
Postcode			2			1	1		
**TREATMENT CHOICE: TOTAL**	**1**	**6**	**11**		**18**	**15**	**10**	**3**	**3**
**Provider characteristics**			**2**		**2**		**2**		**2**
Individual									2
Postcode			2				2		
**Non-surgical Treatment**		**1**	**1**		**2**	**2**			**1**
Individual									1
Postcode		1	1			2			
**No Surgery**			**1**		**1**		**1**	**1**	
Individual								1	
Area - Not defined			1				1		
**Surgery - Breast Conserving**		**1**	**2**		**3**	**5**			
Individual						2			
CD			1			1			
Postcode		1				1			
Area - Not defined			1			1			
**Surgery - Mastectomy**		**2**	**2**		**4**		**6**		
Individual							2		
CD			1				1		
Postcode		1					1		
Municipality		1					1		
Area - Not defined			1				1		
**Surgery - Reconstructive**	**1**		**3**		**4**	**8**			
Individual	1					5			
CD			1			1			
Postcode			1			1			
Area - Not defined			1			1			
**Post-treatment Surgery**		**1**			**1**			**1**	
CD		1						1	
**Treatment Intensity before death**		**1**			**1**		**1**	**1**	
Individual								1	
CD		1					1		
**SURVIVAL: TOTAL**		**6**	**2**	**1**	**9**	**7**		**5**	
Individual						2		1	
CD		2	1					3	
LGA		2				2			
SLA		2	1	1		3		1	
**MORTALITY: TOTAL**		**7**	**2**	**1**	**10**	**2**	**6**	**5**	
Individual						1	1	1	
CD			1				1		
LGA		5					3	2	
SLA		1	1	1			1	2	
Area - Not defined		1				1			
**GRAND TOTAL**	**12**	**31**	**21**	**3**	**67**	**37**	**35**	**32**	**3**

*Note: NS^* Non-significant finding reported by authors, *NR#* Findings not reported by authors

#### Never screened

Three findings produced eight relationships between individual SES and the likelihood of never having had a mammogram. The weight of evidence, four relationships, was suggestive of a non-significant relationship between never screened and education and private insurance status [34], and, income and employment [35]. Negative relationships were also shown, with women who had lower education levels [36], without private health insurance [35] and who resided in more disadvantaged areas [36] being more likely to never have had a mammogram. Conversely, those with higher individual income (> $40,000) [34] were more likely to never attend screening.

#### Recent screening

Six findings described the relationship between individual level indicators of SES and attending recent screening with the weight of evidence, nine relationships, suggesting non-significance. The majority of studies focussed on employment [37–40] and education [37, 38, 41] as indicators of SES. There was limited evidence found for positive associations between recent screenings and higher income [42] and having private insurance [39]. A similar number of relationships were found for a negative association between recent screenings and higher educated women [39, 40]. In one study, the positive associations found between the two indicators were reported to be non-significant [38]. No area or multilevel studies were undertaken.

#### Overdue

Five findings examined overdue screening, again with mixed results. One study reported that higher education status and income level (≥$40,000) as well as having private health insurance were positively associated with overdue screening [34]. Whereas negative associations were reported for indicators of lower education [36], having no private health insurance and a low income (less than $20,000) [35]. One area level study found women residing in more disadvantaged areas had greater likelihood of being overdue in screening [36]. Non-significant relationships were also reported for employment status [35, 43] and area based disadvantage after adjusting for Indigenous and ethnic status at the postcode scale [44].

#### Access to services

We found only one study [45] which reported that women living in disadvantaged areas (measured at the fine area level scale) had greater access to mammography facilities than their advantaged counterparts. However, the magnitude of the difference was not tested for significance.

### Incidence

Five area-based findings examined the relationship of SES (the majority using IRSD) and incidence of breast cancer using state based cancer registries. Four utilised the same cancer registry (New South Wales Cancer Registry) and reported higher incidence of cancer for women who lived in high SES areas when compared to low SES areas [46–49]. This was apparent in major city populations [46, 47], in inner regional areas [46] and using two different area indicators of SES [49]. This positive relationship also held across different scales of analysis, from CD to SLA at the area level (Table 1). One study, however, found an increased risk of metastatic breast cancer was associated with living in areas of lower SES [50]. While data was not shown, difference in incidence by SES measurement type was slightly greater for quintiles defined by the IRSD in comparison to the unemployment rate [49].

### Diagnosis

Ten findings highlighted the individual and area level relationships between SES and diagnosis of breast cancer. Overall, the weight of evidence was towards a negative relationship but this varied within three classifications: stage, procedures and results.

#### Stage

Seven findings found a negative association between SES and the stage of diagnosis. The majority were area based studies where women in living in low SES areas were more likely to be diagnosed with large, advanced staged (distant) tumours [51, 52] compared to those in higher SES areas, who were more likely to be diagnosed with smaller, less advanced staged (localised and regional) tumours [49, 53]. However, non-significance was found between SES and advanced disease stage at the area level [49]. Controlling for the effect of individual SES characteristics (occupation), a multilevel study [54] showed that individual level employment type (blue collar in reference to professionals) and women who lived in the most disadvantaged areas were more likely to be diagnosed with advanced breast cancer. These findings were consistent across different scales of area level analysis (Table 1). One study reported a non-significant relationship between individual level education (after adjustment) and area based disadvantage in distant reoccurrence [49].

#### Diagnostic procedure

One finding reported the significance of individual and area based differences in diagnostic procedures by SES. Women living in low SES areas (scale of area was not defined) were twice as likely to receive open biopsy for a diagnostic procedure as opposed to fine needle aspiration (which was more likely in higher SES areas) and core biopsy [55]. No trend was found when comparing health insurance status.

#### Diagnostic results

Two findings showed mixed results between pathologic test results to identify early invasive cancers and area (postcode) level SES. Significance was dependent on whether the variable analysed was in nominal or ordinal forms. A triple negative result was less common for oestrogen, progesterone and HER2 receptors in women residing in higher SES areas (nominal) but was insignificant as an ordinal variable [56]. Bilateral synchronous lesions were less common in women residing in lower SES areas (ordinal) but were non-significant as nominal variables [56].

### Treatment choice

Eleven findings described SES in relation to different types of treatment received for breast cancer. The majority of studies [56–62] utilised finer area scales of analysis (CD, postcode) (Table 1) and the IRSD as the indicator of SES. The weight of evidence leaned slightly towards a positive association, but significance of these findings differed across the eight areas of treatment classified by the treatment literature (Table 1).

#### Provider characteristics

Two findings investigated individual and area level indicators of SES and provider characteristics in the treatment of early invasive breast cancers [56]. Disadvantaged women had greater odds of being treated in inner regional and remote centres. This interaction between SES and remoteness highlighted that they were 20% less likely to be treated by more experienced high annual case load surgeons. Women living in disadvantaged areas had lower odds (around 20%) of being referred for clinical surveillance (asymptomatic referrals). The significance of these relationships with insurance status was not reported.

#### Non-surgical treatments

Women living in the highest socioeconomic areas with early invasive cancers were more likely to receive ovarian ablation [56] and post-operative radiotherapy following breast conserving surgery [59] when compared to those living in the lowest SES areas. The significance of the relationship with insurance status and ovarian ablation was not reported [56].

#### No surgery

No surgery was the most common outcome for women without private health insurance or those living in low SES areas for early breast cancer [55].

#### Surgery – breast conserving

Breast conserving surgery was the most likely procedure for those with private health insurance [55, 63] and those living in high SES areas [55, 59, 63]. This result did not vary across the individual and finer area levels of SES indicators analysed.

#### Surgery – mastectomy

All findings investigating mastectomy showed that this was the most likely procedure for women without private health insurance [55, 63] and living in low SES areas [59, 64]. This relationship was consistent across individual and area level indicators of SES, differences in area scales and variations in indicators definitions. Here, education [64] and a composite indicator of average education, employment and income [59] were used instead of the IRSD.

#### Surgery – reconstructive

Reconstructive surgery following mastectomy was more likely for higher educated women [65] or those with private health insurance [55, 57, 60, 65]. Reconstruction following mastectomy was also higher in women residing in high SES areas [55, 57, 60]. This result was consistent across finer scales of area SES analysis.

#### Post-treatment surgery

Fine scale area based SES was not associated with further breast conserving or mastectomy surgery [62].

#### Treatment intensity before death

Women living in highly disadvantaged areas had a significantly increased rate of hospitalisation in the last year of life while having private health insurance was found to be non-significant [61]. No significant differences were shown in the second and third year prior to death.

### Survival

Nine findings described the effect of SES on survival. The majority of these used area level SES indicators with two studies [66, 67] using individual and area level indicators and one undertaking multilevel analysis [68].

Women in professional occupations [67, 68] and those living in the most advantaged areas [68–72] had higher survival. Two studies also reported this trend but reported non-significance at the individual [66] and area level [66, 73] after statistical attenuation. Non-significance was also reported at the area level [67, 74]. However, this result was apparent for 5-year unadjusted survival estimates but changed for 10 year estimates where survival was lower for more women living in disadvantaged areas [67].

Comparison of the scale of area analysis used showed that the significant results found were based on coarser scale of analysis. Non-significant relationships were found at the finer scales. Comparison of differences in area level definitions of SES at similar scales showed no difference in the results found, for example, the comparison of IRSD to the index of education and occupation [70, 71] and the unemployment rate [74].

### Mortality

Ten findings, the majority using area level indicators, examined the effect of SES on mortality. Conflicting results were found at the individual level. Relative risk of mortality was higher (in descending order) for blue and white collar and professional occupations when compared to not in the labour forces [67] while a multilevel study found that professionally employed woman had a higher rate of mortality [75]. Alternatively, having private health insurance had no relationship with mortality [58].

Mixed results were also found at the area level, with five findings reporting negative associations indicating that those living in the most disadvantaged areas had a greater risk of dying following diagnosis of breast cancer than those in less disadvantage areas [53, 58, 69–71]. However, four findings reported non-significant relationships [47, 67], one of which was undertook multilevel analysis [75] and one after statistical adjustment for degree of spread of diagnosis [76]. These findings used similar coarse levels of area scale for their analyses with only one using a finer level of analysis [58]. One finding with area scale not defined reported contradictory evidence, with 10% higher mortality in women living in high SES areas compared to those in low SES areas [77]. This study used a different definition of area based SES than the IRSD which was more commonly used. Three other findings also used different definitions showing negative [70, 71] and also non-significant results [76].

## Discussion

**Fig. 2:**
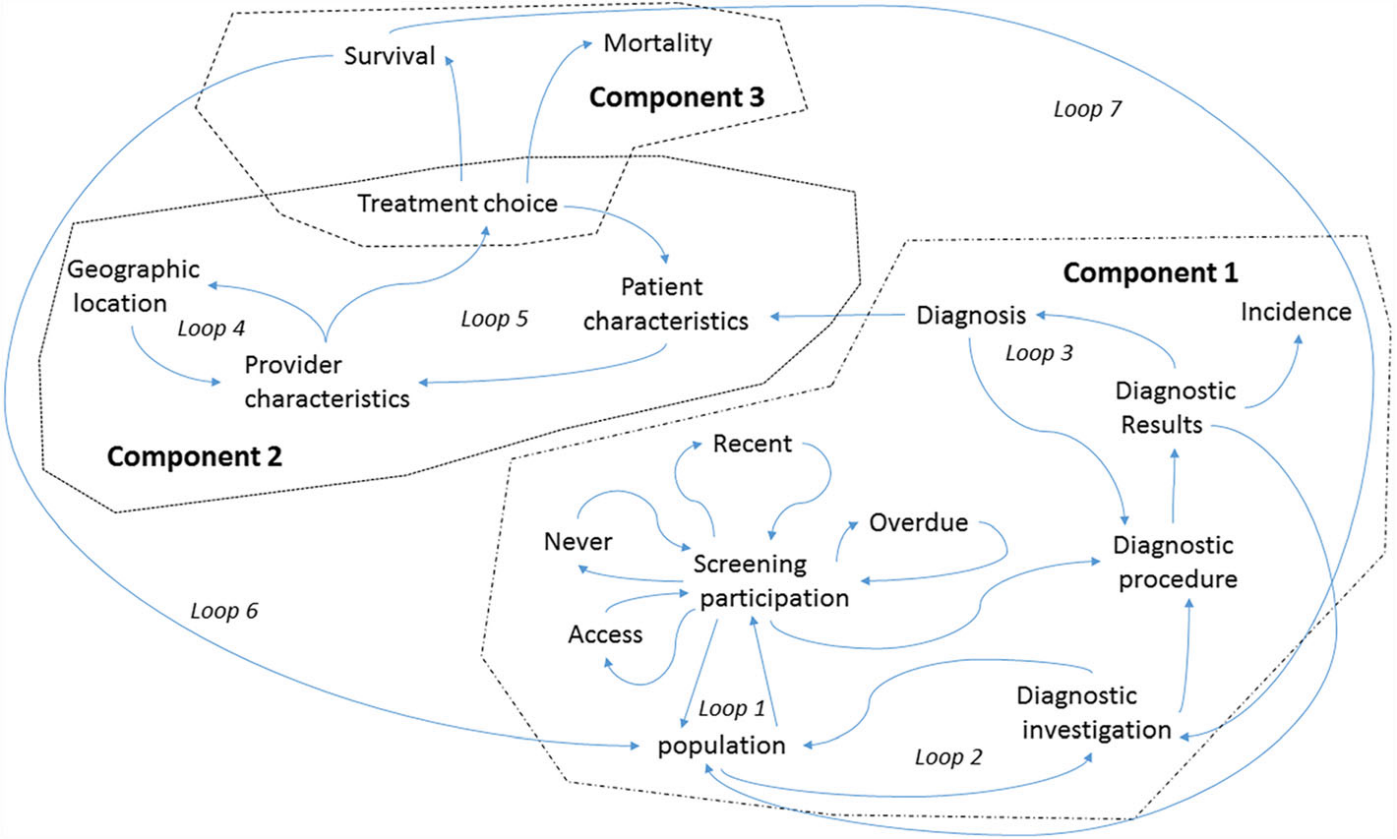
Feedback diagram highlighting the interaction of elements along the breast cancer continuum both within and across the three components

To illustrate the interrelatedness of the SES findings, a feedback diagram (Fig. 2) is proposed. The diagram enables us to contextualise the findings of this review as interrelated components and elements within a larger, complex system. The model consists of three components: Component 1 represents the interaction of the mammography, diagnosis and incidence elements, Component 2 embodies the treatment interactions once women have been diagnosed, and Component 3 signifies survival and mortality relationships.

### Component 1: mammography, diagnosis and incidence

The outcomes of Component 1 are diagnosis and incidence, and the findings for Australia were similar to international studies showing a higher cancer incidence for women who live in high SES areas [9, 19] and a greater likelihood of late stage diagnosis for more disadvantaged women [78, 79].

Figure 2 shows two potential paths from the population to diagnosis with returning arrows from Screening participation, Diagnostic investigation and Diagnostic results elements indicating no cancer diagnosed. The paths illustrate either the choice to participate in the national screening program (Loop 1) or the use of mammography as a diagnostic tool for women who present with symptoms (Loop 2). Loop 2 can either relate to those women who present with symptoms within the 2 year period between screenings or those who present with symptoms and do not participate in screening. Internationally, the higher participation of advantaged women in Loop 1 (screening) [5, 80] has been identified as a possible explanation for the SES differences in diagnosis and incidence even in countries where financial barriers are absent [81]. We found little evidence of this with never having had a mammogram, participating in a recent screening or being overdue for a screening having relatively small associations between individual level SES. However, there are a number of methodological issues that should be considered. Firstly, the individual fallacy bias may be apparent as the majority of studies are based on individually defined SES (compositional) indicators. This means that the contribution of area-based SES contextual factors have not been incorporated into the majority of the evidence base. Secondly, the study years examined also varied with the majority of studies published in the early – mid 2000’s. It is possible that the results of past literature do not reflect the current trends, particularly since recent evidence has suggested that the concerted effort to reach disadvantaged groups has been effective [67]. Accessibility to screening facilities has also been a factor in the equitable delivery of services. We found only one year 2000 metropolitan based study [45] that reported that low SES women had greater access to mammographic screening. The lack of published research of potential trends within Australia represents a significant gap in our understanding of equitable service provision, particularly as access is regularly cited as a major barrier to influence participation [82].

Loop 2 reflects the underlying pattern of differences in health and health service utilisation between women with low and high SES characteristics. Evidence suggests poorer health is experienced by those in lower SES areas [83] leading to higher general practitioners visits [84]. However, this increased visitation does not lead to increased clinical surveillance for breast cancer by asymptomatic referrals [56]. This may partially explain why our findings showed that incidence was lower and stage of diagnosis was more advanced in women with low SES. No studies included in this review examined the influence of SES on the early phase of cancer investigation for women who present with symptoms and use mammography as a diagnostic investigation tool. Alternatively, the utilisation of medical specialists more generally has been found to be higher in high SES areas [85] after initial investigations have been carried out. This difference in utilisation may point to why, we found that incidence was higher but the stage of diagnosis was lower in high SES compared to low SES women.

Relating the diagnosis outcome to the diagnostic procedures element through Loop 3 may also explain some of the SES differences found in the choice of procedures used. Here, the more advanced stage of cancer found in women from low SES areas meant that the diagnostic procedures administered were more invasive and certain diagnoses results (triple negative result) were more prevalent. Similar findings have been reported internationally [79, 86, 87].

### Component 2: treatment choice

Australian treatment choice results were similar to international findings, with women living in deprived areas having reduced odds of surgery and radiotherapy [86], breast reconstruction surgery [88, 89] and higher rates of non-conservative surgery [90] and mastectomies [86]. Fig. 2 illustrates within Component 2 that treatment choice is a result of the interaction between patient and provider characteristics which are presented as two reinforcing loops. Loop 4 highlights the interaction between provider characteristics such as type and quality of care received and geographic location. Australia has a vast land area, therefore geographic location is particularly influential in terms of SES and equitable service provision. For example, one national study [56] reported that patients from lower SES areas were more likely to live in more remote areas and as such were more likely to be subject to the treatment choices available in regional than major city hospitals. Additionally, in more remote areas, there is a greater odds of receiving treatment from lower caseload surgeons which has implications for the choices made for initial patient management, treatment [91, 92] and outcomes [91, 93].

Loop 5 incorporates the outcome of treatment choice which is predicated on the complex interaction between the outcomes of Loop 4’s provider characteristics in terms of what treatment choices are available and the interaction with patient characteristics such as age and the diagnosis inherited from Component 1 (Fig. 2). This inheritance may explain SES differences in the treatment choices undertaken. For surgery choices, we found that mastectomies were more likely conducted in women with low SES who were found with more advanced stages of the disease. Alternatively, women with high SES were more likely to have breast conserving surgery possibly reflecting the less advanced stage of diagnosis, or standard practice that tumours less than 20 mm in diameter are treated with breast conserving surgery [94]. Patient characteristics incorporated into Loop 5 may also explain SES differences across different stages of treatment. For example, the most common initial treatment for women with low SES and with early stage cancer was no surgery rather than breast conserving therapy and ovarian ablation procedures. Furthermore, those women with low SES and receiving end stage treatments were less likely to have post-operative radiotherapy and breast reconstruction surgery. Some possible explanations for the choice for less conservative treatments may reflect their difficulty in weighing up a range of potential options and side effects because of their lower education levels. Additionally, the potential additional indirect costs and disruptions to family and work life associated with additional treatments may also be unpalatable especially to those on lower incomes or in certain types of employment.

One major limitation in the Component 2 subsystem is that the initial establishment of association between geographic location (remoteness) and SES has had limited exploration specifically in relation to women diagnosed with breast cancer. Apart from the national study advocating SES differences in remote areas, two state based studies using the same cancer registry reported no association between remoteness and SES [54, 68]. Our review also identified several other papers [59, 73] that focussed on hospital factors and remoteness as key influencers of outcomes but none investigated remoteness as a relationship with SES. The small evidence base and conflicting results demonstrates a much needed future direction to investigate if SES gradients exist across each region’s urban, rural and remoteness classification [54] in reference to the treatments chosen.

No studies were found on the relationships between SES and surgical outcomes, post-surgical complications, length of stay and waiting times in diagnosis and treatment stages which are used as surrogates for the quality of treatment received. These issues have been reported internationally [3, 86, 95] and need to be explored further.

### Component 3: survival or mortality

Component 3’s elements of survival and mortality represent the outcomes from the treatment choice undertaken in Component 2. Two feedback loops are proposed, Loop 6 which represents a movement of a women, surviving cancer, back to the population. Loop 7 represents a path to diagnostic investigation for recurrences. Non-significant relationships for individual (after adjustment) or area level SES relationships were reported for distant recurrences [49] while no evidence was available for SES relationships with other recurrences.

Survival and mortality elements combined with Component 1’s element of incidence are essential population based indicators for public health and cancer control [96]. Just over half the findings reported higher rates of survival for women in high SES areas with others reporting non-significance. Less evidence of an SES gradient was found for mortality, with six findings reporting negative relationships, four reporting non-significant associations and two positive relationships. Similar findings have been reported internationally for survival [79, 97–99] and mortality [4, 19]. Interactions within the components of the model may suggest a greater influence of SES than the current evidence suggests. For example, with a higher incidence of small localised breast tumours in women with high SES we may expect a greater likelihood of survival and less mortality while a lower incidence of more advanced stage of cancer in low SES women should point to a lesser likelihood of survival and greater mortality. However, this isn’t supported by the survival and mortality literature. It is possible that the influence of SES across the components is moderated by the choice of treatments in Component 2 which in one study was found, in general, to be similar across the SES gradient [56]. Additionally, even with different types of surgery which was also predicated by SES i.e. breast conserving surgery versus mastectomy, survival has been shown to be similar across different diagnosis stages [100, 101].

### Methodological issues and limitations

This review focused on Australian women as the target population, which includes some women with Indigenous heritage and other ethnicities as part of the general population, however, our exclusion of papers that focused only on this population, may have influenced our findings. Indigenous women living in Australia are more likely to be of lower SES, have greater remoteness, and experience worse outcomes at all stages of the cancer trajectory than women of non-Indigenous heritage [102]. Across the breast cancer continuum, comparison of Indigenous to non-Indigenous women have found that they are more likely to be disadvantaged [103], are less likely to participate in breast cancer screening and rescreening [104], have more advanced stages of diagnosis and more likely to die prematurely [103–105]. Interestingly, the evidence for uptake of treatment is mixed. One study finding has reported no difference in treatment patterns [103] while another found Indigenous women were less likely to receive surgical treatment [105]. When surgery was undertaken the choice of treatment also differed with mastectomy a more common procedure than complete local excision [104]. While Indigenous women make up a small proportion of the total Australian population, they experience poorer cancer outcomes. Future research needs to improve our understanding of this inequity across the cancer continuum.

Across the range of studies we found that issues of indicator type and composition, and to a lesser extent the modifiable areal unit problem (MAUP), were not significant across the majority of evidence. For indicator types and composition, this result was more to do with the fact that the majority of studies used the Index of Relative Socio-economic Disadvantage (IRSD) to represent area level SES. Where studies used different measures, they were usually based on income, education or occupation and produced similar relationships to using the IRSD. This suggests that differences in the selection and composition of indicators were not an issue in result differences. We did find small evidence for the MAUP in the evidence gleaned for survival where results at the fine scale were non-significant compared to positive associations found at coarser scales. However, a recent study [106] has provided statistical evidence that changes in scales of analysis showed little difference in survival rates.

This study may have potential limitations that bias the evidence reported through the databases which were searched and the exclusion of the grey literature. Inclusion of the grey literature may have provided more evidence as our restriction to peer-reviewed studies are more likely to report significant findings. The evidence shown in this study does have a publication bias, with over a third of the evidence obtained coming from studies investigating the New South Wales’ population, 32% of the Australian population. This bias was significant in the survival and mortality evidence base where studies from this geographic area made up the majority of results. Given the small number studies for some stages across the continuum the strength and direction of the relationships reported needs to be interpreted with caution. Further, a longitudinal view of the SES gradients experienced by women across the continuum is needed. While the number of studies found in each stage is a limitation it also represents areas where more research should be undertaken to create a larger sample size ideally across other geographic areas thus decreasing the publication bias found.

There was a lack of multilevel analysis studies, with only three found across the continuum. Our evidence does suffer from the methodological issues of individual fallacy for screening studies and ecological fallacy in other outcomes along the continuum. Given that the results of the three multilevel studies corroborated with the results found in other studies i.e. in the issues of diagnosis [54], survival [68] and mortality [75], we can have some confidence in the evidence base derived from the alternative study designs. Undertaking multilevel analysis in the area of screening provides the easiest opportunity as individual level data is already collected and application of area level SES is quite arbitrary if residential location is available. These types of studies are key to understanding the strength of the compositional (individual) or contextual (area) effects of SES providing evidence for what type of interventions can be applied. This evidence can inform policy decisions with individual level SES factors driving changes to broad policies at the population level. Evidence of area level SES interactions could mean a more targeted approach to resource allocation [107], applying different strategies for the same groups in different areas [108] or the allocation of resources across multiple levels [109] of the continuum.

We found disparities along the continuum. Our feedback diagram provides a way to summarise the evidence base and examine relationships within a wider integrated framework. Considering the associated economic costs in future research will help quantify the impacts on quality of life for people and their families, the health care system and society.

## Conclusion

In a universal health system such as that in Australia, evidence of an SES gradient exists, however, the strength and direction of this relationship varies along the breast cancer continuum. This is a complex relationship and the heterogeneity in study design, the SES indicator selected and the scale they represent further complicates our understanding of its influence. More complex multilevel studies are needed to better understand these relationships, the interactions between predictors and to reduce biases introduced by methodological issues.
